# 3D gait analysis in children using wearable sensors: feasibility of predicting joint kinematics and kinetics with personalized machine learning models and inertial measurement units

**DOI:** 10.3389/fbioe.2024.1372669

**Published:** 2024-03-20

**Authors:** Shima Mohammadi Moghadam, Pablo Ortega Auriol, Ted Yeung, Julie Choisne

**Affiliations:** Auckland Bioengineering Institute, The University of Auckland, Auckland, New Zealand

**Keywords:** 3D gait analysis, inertial measurement units, machine Learning, pediatric, joint kinematics, joint kinetics

## Abstract

**Introduction:** Children’s walking patterns evolve with age, exhibiting less repetitiveness at a young age and more variability than adults. Three-dimensional gait analysis (3DGA) is crucial for understanding and treating lower limb movement disorders in children, traditionally performed using Optical Motion Capture (OMC). Inertial Measurement Units (IMUs) offer a cost-effective alternative to OMC, although challenges like drift errors persist. Machine learning (ML) models can mitigate these issues in adults, prompting an investigation into their applicability to a heterogeneous pediatric population. This study aimed at 1) quantifying personalized and generalized ML models’ performance for predicting gait time series in typically developed (TD) children using IMUs data, 2) Comparing random forest (RF) and convolutional neural networks (CNN) models’ performance, 3) Finding the optimal number of IMUs required for accurate predictions.

**Methodology:** Seventeen TD children, aged 6 to 15, participated in data collection involving OMC, force plates, and IMU sensors. Joint kinematics and kinetics (targets) were computed from OMC and force plates’ data using OpenSim. Tsfresh, a Python package, extracted features from raw IMU data. Each target’s ten most important features were input in the development of personalized and generalized RF and CNN models. This procedure was initially conducted with 7 IMUs placed on all lower limb segments and then performed using only two IMUs on the feet.

**Results:** Findings suggested that the RF and CNN models demonstrated comparable performance. RF predicted joint kinematics with a 9.5% and 19.9% NRMSE for personalized and generalized models, respectively, and joint kinetics with an NRMSE of 10.7% for personalized and 15.2% for generalized models in TD children. Personalized models provided accurate estimations from IMU data in children, while generalized models lacked accuracy due to the limited dataset. Furthermore, reducing the number of IMUs from 7 to 2 did not affect the results, and the performance remained consistent.

**Discussion:** This study proposed a promising personalized approach for gait time series prediction in children, involving an RF model and two IMUs on the feet.

## 1 Introduction

Children’s walking patterns are distinctive from adults and evolve with age (Cigali et al., 2011; Senden et al., 2023). At a small age, gait tends to be less repetitive and will differ from those of adults (Ganley and Powers, 2005; Jain et al., 2016), emphasizing the need to build normative data for a pediatric population (Ganley and Powers, 2005). The differences in school-aged children’s walking patterns are often attributed to their significant musculoskeletal changes (Bari et al., 2023), given that they are in a critical stage of growth and development (Onis et al., 2007). A recent study (Bach et al., 2021) suggested that the degree of gait maturity does not always directly relate to the chronological age of the child. This finding underscores the complexity of assessing gait development in children. Moreover, it's been shown that, compared to adults, young individuals exhibit more variable kinematic patterns when performing repetitive movements (Kuhtz-Buschbeck et al., 1996). Additionally, the observed variability in Electromyography (EMG) waveform within-session for children exhibited approximately twice the variability of EMG signals (muscle activation level) for adults (Granata et al., 2005), affecting their joint kinematics and kinetics within a single session.

Three-dimensional gait analysis (3DGA) is a valuable tool for understanding a child’s gait pattern and how it compares with normative data of typically developed children’s gait (Ito et al., 2022; Bari et al., 2023). The insights gained from 3DGA in children affected by lower limb movement disorders serve as a foundation for clinical assessment to target personalized treatment and improve their walking patterns (Bari et al., 2023). Considering the unique challenges and broad spectrum of motor impairments in this population, addressing developmental challenges requires a tailored approach. The current gold standard for performing 3DGA involves Optical Motion Capture (OMC) along with force plates due to its high accuracy and robustness (Chester et al., 2005). However, the high cost of OMC systems and the time-consuming data post-processing needed lead to long waitlists for patients and sometimes long-distance travel for families coming from rural areas (Aminian and Najafi, 2004).

Wearable sensors like Inertial Measurement Units (IMUs) are potential alternatives to the OMC system, enabling the potential to capture 3DGA in rural areas and natural environments (Gurchiek et al., 2019). Unlike the OMC systems, IMUs are inexpensive, small, and lightweight and can be used outside the clinic by wearing them or attaching them to the children’s limbs or pelvis (Aminian and Najafi, 2004). Although IMU sensors are very promising in motion analysis, challenges such as time-increasing drift errors, which result in less accurate estimations, still need to be overcome (Aminian and Najafi, 2004). Moreover, traditional approaches, such as sensor fusion algorithms (Sabatini, 2006; Madgwick et al., 2011), as well as the tool package OpenSense (Al Borno et al., 2022), require placing an IMU on each body segment for accurate kinematics calculations and functional calibration.

The challenges associated with processing IMU data in adult populations have been addressed in previous studies (Findlow et al., 2008; Luu et al., 2014; Dorschky et al., 2020; Giarmatzis et al., 2020; Lim et al., 2020; Stetter et al., 2020; Mundt et al., 2021; Sharifi Renani et al., 2021; Tan et al., 2022; Moghadam et al., 2023a) by implementing Machine learning (ML) models. While each of these studies utilized a combination of IMUs and ML techniques, their focuses varied: some concentrated on predicting joint kinematics (Findlow et al., 2008; Luu et al., 2014; Dorschky et al., 2020; Sharifi Renani et al., 2021; Tan et al., 2022), som on joint kinetics (Giarmatzis et al., 2020; Stetter et al., 2020), and few on both kinematics and kinetics prediction (Lim et al., 2020; Mundt et al., 2021; Moghadam et al., 2023a). These ML models can establish a direct relationship between the IMUs’ data and OMC derived gait time series such as, joint kinematics, joint kinetics, and muscle forces (Moghadam et al., 2023a). Prior research indicated the efficacy of this approach in adult populations, demonstrating highly accurate results with low errors during the personalized model (tested on the same individual used for training). Additionally, reliable estimations were yielded using generalized models (tested on new participants not included in the training set), even in scenarios with limited dataset availability. Among various ML models developed for the adults population, artificial neural networks (ANN) have been widely utilized for predicting gait time series. However, there is a limited body of literature exploring alternative data-driven models that may demand smaller datasets while achieving comparable results to ANNs. Building on this context, in a prior study, we demonstrated that Random Forest (RF) models can yield results comparable to more intricate machine learning models such as Convolutional Neural Networks (CNNs) for 3D Gait Analysis (3DGA) in adults (Moghadam et al., 2023a). Given the greater heterogeneity in children’s gait, it will be interesting to explore whether RF or CNNs can be applied to a pediatric population with similar performances.

The primary focus of existing ML models for 3DGA in children lies in gait classification (Kamruzzaman and Begg, 2006; Zhang et al., 2009; Zhang and Ma, 2019; Choisne et al., 2020; Khaksar et al., 2021) rather than the development of models for predicting gait time series. There are only a handful of studies focused on predicting children’s gait using ML techniques (Kwon et al., 2012; Vigneron et al., 2017; Morbidoni et al., 2021; Kolaghassi et al., 2022; Kolaghassi et al., 2023). A research group used EMG sensors’ signals to predict children with cerebral palsy (CP) knee moment and achieved high correlation coefficients between 0.71 and 0.93 for different participants (Kwon et al., 2012). Another study proved the feasibility of using neural networks in predicting gait events from surface EMG signals in hemiplegic cerebral palsy (Morbidoni et al., 2021). Other studies have employed ML techniques to estimate one-step-ahead gait trajectories to control lower-limb robotic devices in children with CP (Kolaghassi et al., 2022; Kolaghassi et al., 2023). However, none of the mentioned studies utilized IMUs’ data to develop the ML model. Given the effective performance of a combination of IMU and ML models in adults, exploring its applicability in a heterogeneous pediatric population would be an interesting avenue for investigation.

It is noteworthy that previous studies have indicated the feasibility of predicting diverse gait time series in adults using a single IMU on the pelvis (Lim et al., 2020) or a pair of IMUs on the shanks (Sharifi Renani et al., 2020; Yeung et al., 2023) or the feet (Gholami et al., 2020). However, given the unique challenges posed by children’s gait, it remains crucial to extend this inquiry to children’s gait analysis by exploring the applicability of using a reduced number of IMUs. A high number of IMU sensors on the body could be impractical in real-world gait analysis, particularly for at-home applications, as it requires high computational power to monitor numerous IMUs (Sivakumar et al., 2019). Therefore, another aspect requiring investigation is to quantify the optimal number of IMUs needed for accurately estimating gait time series in children.

Therefore, this study aimed to assess the feasibility of leveraging data from IMUs to construct ML models for predicting gait time series in school-aged children. This goal was pursued through three key objectives. Firstly, we sought to explore whether personalized and generalized ML models for predicting gait time series in children could demonstrate comparable efficacy to their adult counterparts. Secondly, an evaluation was conducted to compare the accuracy of two distinct ML models–the multi-output RF and CNN models–for predicting gait time series in children. The final objective centered on exploring the potential of placing a singular IMU on each foot, as opposed to employing seven IMUs distributed across all lower limb segments.

## 2 Materials and methods

### 2.1 Participants

Seventeen typically developed (TD) children (9 Females, 8 Males; age = 10.5 ± 2.8 yr [6:15]; height = 147.2 ± 16.9 cm [119:174]; weight = 37.1 ± 11.7 kg [19.7:56.9]) were recruited for this study. Each child’s legal guardian provided informed consent prior to data collection. The research strictly adhered to ethical principles outlined in the Helsinki Declaration and received approval from the University of Auckland (New Zealand) human participant ethics committee (reference number 021615).

### 2.2 Data collection

**FIGURE 1:**
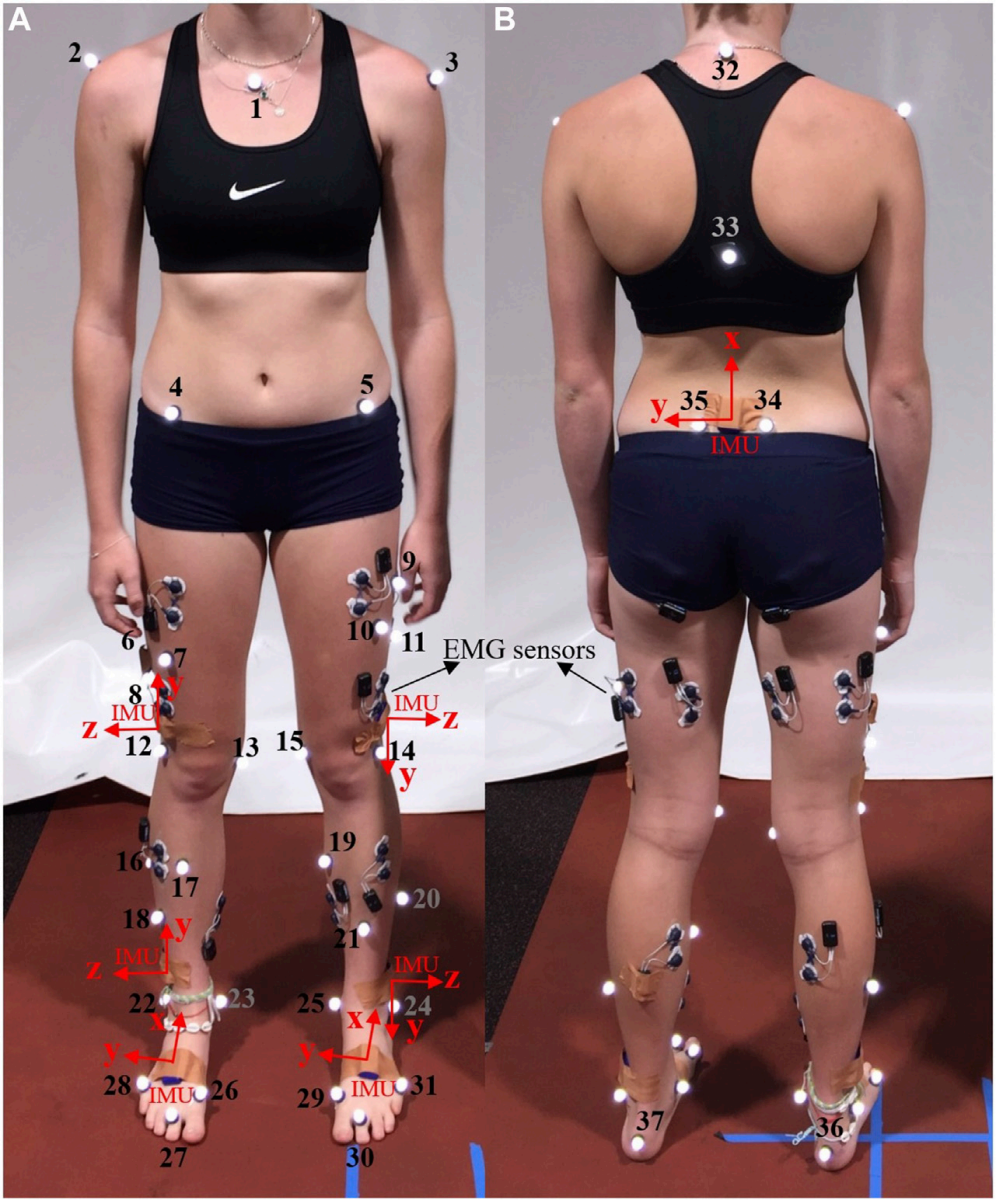
Pictures of the front **(A)** and back **(B)** of a participant, illustrating markers’ placement (numbered in the image) and sensor locations (IMU axes depicted in red). The study did not utilize data from the Electromyography (EMG) sensors.

OMC, force plates, and IMUs data were recorded while each participant completed one static and a minimum of 15 over-ground walking trials for over 20 m at their self-selected speed. For the initial five participants, we affixed 37 reflective markers, indicated by numbers 1 to 37 in Figure 1, on their body segments. Subsequently, a paper by Bakke and Besier (2022) from our lab suggested a streamlined marker set, removing markers 6, 7, 8, 9, 10, 11, 18, 21, 32, and 33, which demonstrated equivalent accuracy in kinematics calculation. For the remaining participants, we adopted this refined marker set with 27 markers. Marker trajectories were traced by a 14-camera optical motion capture system (Vicon Motion Systems Ltd., United Kingdom) at a sampling frequency of 100 Hz for all trials. In addition, seven IMU sensors [Blue Trident, Vicon IMeasureU Ltd. (NZ)] were secured on the participants’ pelvis (between left and right posterior superior iliac spine markers), thighs (1 cm above the lateral aspect of the patella), shanks (1 cm above the lateral aspect of the ankle), and feet (on the dorsal surface) as shown in Figure 1, and recorded three axes of angular velocity and linear acceleration at 2 kHz. Ground reaction forces (GRFs) were acquired at 2 kHz from three force plates (Bertec, Columbus, Ohio) embedded in the gait lab floor. The Vicon Nexus software (version 2.12) was used to collect and synchronize marker trajectory, GRF, and IMUs data and subsequently to reconstruct markers’ trajectories.

### 2.3 Data processing

After extracting data as C3D files from Nexus, MOtoNMS, a Matlab Motion data elaboration toolbox for neuromusculoskeletal applications (Mantoan et al., 2015), was used to filter marker trajectories and ground reaction forces (GRF) using a Butterworth fourth order, 8 Hz low pass filter. Then MOtoNMS was employed for rotating and aligning the lab coordinate system to the OpenSim coordinate system, where X, Y, and Z-axes correspond to the frontal, transverse, and sagittal planes, respectively. Additionally, MOtoNMS was utilized to determine hip joint center (HJC) locations using Harrington regression equations from static trials for scaling in OpenSim (Harrington et al., 2007).

A musculoskeletal model was created for each participant by linearly scaling OpenSim gait 2392 model (Delp et al., 2007), which is a generic adult model. The Gait 2392 model is a detailed biomechanical representation, featuring 23 degrees of freedom and 92 musculotendon actuators. Within this model, the pelvis and hip joints offer three rotational degrees of freedom each, allowing for movements in the three planes of motion. The pelvis in Gait 2392 allows for movements such as tilt, obliquity, and rotation in the transverse plane, facilitated by its complex structure of joints. The hip joint is characterized as a ball-and-socket joint, enabling motions such as flexion/extension, adduction/abduction, and internal/external rotation. The knee model is a simple hinge joint with one degree of freedom allowing for flexion/extension. Additionally, the ankle (allowing for ankle dorsi/plantar flexion) and subtalar (allowing for ankle inversion/eversion) joints are simulated as frictionless revolute joints. The scaling tool in OpenSim (version 3.3) aligns virtual markers on the generic model with those placed on specific anatomical landmarks of the participant’s body during the static trial. The HJCs calculated by MOtoNMS were used to scale the femur. The kinematics and kinetics of the lower limb joints, including the pelvis (3 DOF), hip (3 DOF), knee (1DOF in the sagittal plane), and ankle (2DOF; sagittal and frontal planes), were calculated for all participants using the inverse kinematics (IK) and inverse dynamics (ID) tools in OpenSim. To estimate joint kinematics and kinetics, we picked two gait cycles from each trial, resulting in a minimum of 30 gait cycles for each participant. The IK tool employs an optimization technique to ensure precise alignment between the virtual markers on the scaled model and the corresponding experimental markers in a least-squares sense (Lu and O connor, 1999; Knudson, 2007). For joint kinetics prediction, we focused on the gait cycles occurring on the force plates to allow for joint forces and moments calculation through the ID tool, which solves the equations of motion (Davis et al., 1991). We excluded trials where the participant’s feet were not entirely within the force plates. Therefore, a variable number of kinetics gait cycles remained for each participant, ranging from a minimum of 8 to a maximum of 18.

This process resulted in a dataset encompassing measurements for 15 joints kinematics and 15 joints kinetics targets, including pelvis tilt, pelvis rotation, pelvis obliquity, hip rotation, hip flexion, hip abduction/adduction, knee flexion/extension, ankle dorsi/plantar flexion, and ankle inversion/eversion joints angles and moments for both legs. Finally, the IMU data were down sampled to 100 Hz to align the data’s frequency with the joint kinematics and kinetics frequency. This also reduces the computational load for feature extraction and machine learning (ML) model construction.

### 2.4 Joint kinematics and kinetics prediction using ML models

**FIGURE 2:**
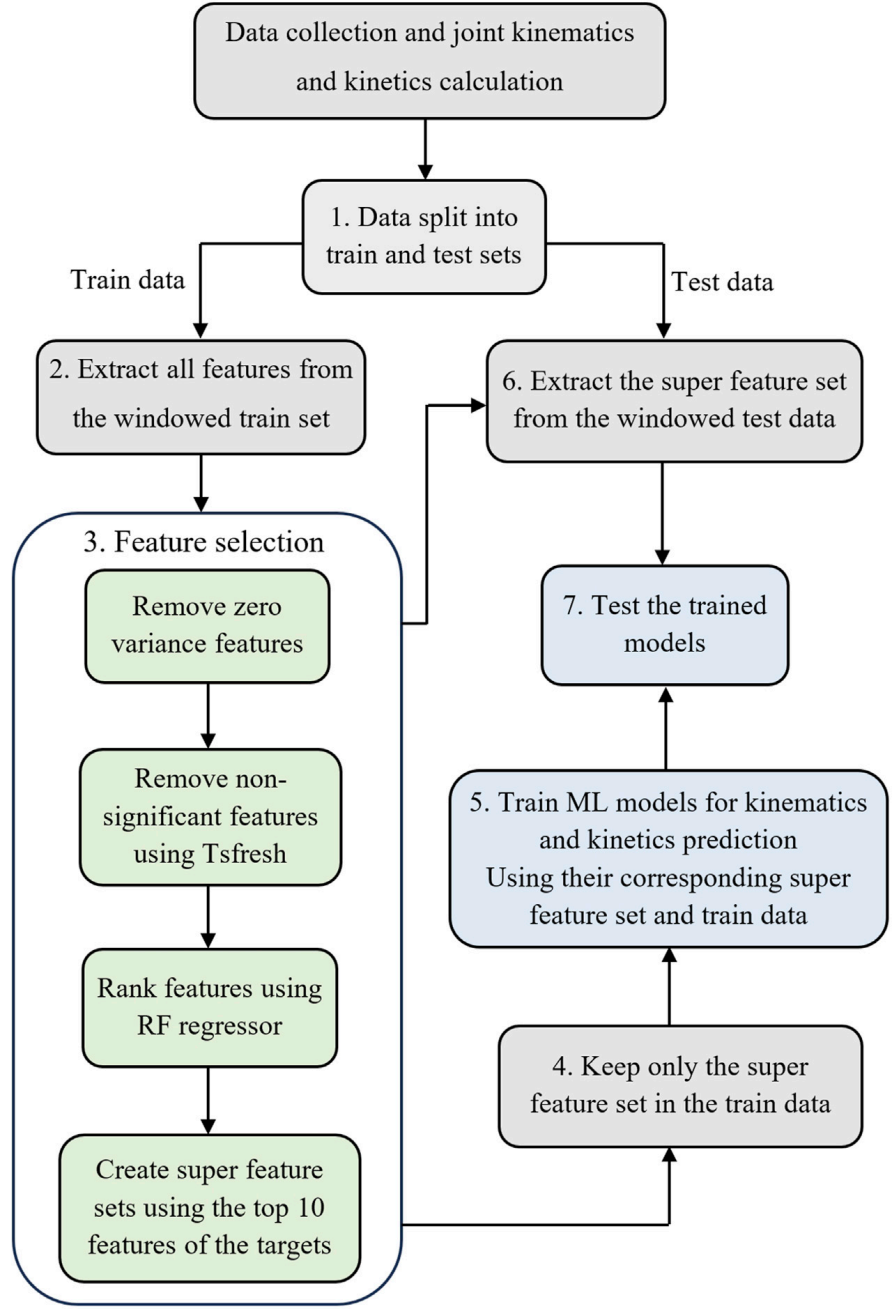
The workflow to develop the ML models. Step 1: Split the data into training and testing sets. Step 2: Window IMU data and feature extraction for the training dataset. Step 3: Feature selection. Step 4: Keep only the selected features in the training set. Step 5: Train ML models using selected features in the training set. Step 6: window IMU data and extract the super feature set (determined in step 3) for the test dataset. Step 7: Test the trained model on the testing set.

After processing data for the 17 participants, a total of 73,364 time points for joint kinematics and 21855 time/data points for joint kinetics were used for the development of ML models. The outlined procedures (Figure 2), including windowing IMU data, feature extraction, feature selection, model development, and model evaluation, were executed as detailed in the subsequent sections.

#### 2.4.1 Training and testing sets

We implemented two distinct data splitting methods to facilitate two types of examinations (Figure 2, Step 1); the first looked at the intra-subject examination accuracy, and the second looked at the inter-subject prediction accuracy.

Intra-subject examination: In this approach, the training dataset consisted of 70% of a participant’s gait cycles, and the remaining 30% of gait cycles were allocated for the testing dataset. A total of 17 training and testing datasets were created to cover all participants and perform the intra-subject examination.

Inter-subject examination: To create training and testing datasets for this examination, we employed a leave-one-out approach for our cohort of 17 participants. The dataset was partitioned to assess the model’s generalization across diverse individuals. During each iteration, one participant’s gait cycles were set aside for testing, while the gait cycles from the remaining 16 participants constituted the training dataset. This process was repeated 17 times, each time excluding a different participant from the training set.

#### 2.4.2 IMU sensors data windowing and feature extraction

In our pursuit of enhancing the accuracy of the learned models and emphasizing the main characteristics of the input data (Laird and Saul, 1994), we adopted a feature engineering technique. From each IMU, we took six time series, encompassing triaxial angular velocity and linear acceleration, to extract features.Thus, we had a total of 42 data vectors from seven IMUs. We organized the input time series data into sequences of consecutive, sliding, and overlapping windows. We selected a window size of 0.75 s, as shown to be the most accurate in predicting gait time series (Moghadam et al., 2023b).

Then, we employed the Tsfresh (Christ et al., 2018) (Time Series FeatuRe Extraction on the basis of Scalable Hypothesis tests) python package to perform feature extraction on the windowed input data (Figure 2, Step 2). This process yielded a feature vector ${\vec{x}}_{i}=\left(\;f_{1}\left(x_{1}\right),f_{2}\left(x_{2}\right),.\;.\;.,f_{m}\left(x_{i}\right)\right)$ for each vector of input data $\left(x_{i}\right)$ . Tsfresh extracted 788 distinct features from each channel of IMU data, resulting in a substantial total of 33,096 features derived from the 42 channels of input data.

#### 2.4.3 Feature selection

The presence of irrelevant and noisy features may considerably reduce the performance of the ML model. The process of removing irrelevant features and selecting the most relevant features is called feature selection (Figure 2, Step 3). We eliminated all zero-variance features to initiate the process of determining the most important features. Then, the Tsfresh feature selector’s built-in function was utilized to remove any non-significant feature, using the Benjamini-Hochberg method (Benjamini and Hochberg, 1995). In the next step, the remaining features were ranked based on their Gini Importance in predicting each target using a Random Forest (RF) regressor (Hasan et al., 2016). Then, the top ten features associated with each target were selected. Our previous findings demonstrated that this selection of 10 features per target yields precise estimations in multi-output models (Moghadam et al., 2023b).

After feature selection, two comprehensive feature sets were constructed, each including 150 features. The first merged all the top features related to kinematics targets, forming the basis for a multioutput ML model dedicated to kinematics prediction. The second feature set put all the top features associated with kinetics targets together to develop a multioutput ML model tailored for kinetics prediction. We retained only the features present in the super feature set from all the extracted features for the training dataset (Figure 2, Step 4).

#### 2.4.4 Non-linear regression ML models

We developed RF and CNN models to assess their accuracy in predicting lower limb joint kinematics and kinetics during gait (Figure 2, Step 5). The hyperparameters for both RF and CNN models were chosen based on previously optimized models (Moghadam et al., 2023a). We employed an RF model comprising 500 trees, each with a maximum depth of 25.

For the CNN model, we used a multi-output architecture with five hidden layers. The selected features were scaled using the Standard Scaler function from the Sklearn library to ensure all variables fell within the same range (between zero and one). Targets were also scaled, and post-prediction, they were rescaled to their original values using the same scaler. The model’s architecture featured an input layer with a size of 150, followed by two convolutional layers, each followed by a max-pooling layer. Both convolutional layers comprised 256 filters with a kernel size of three and employed a “relu” activation function. The max-pooling layers had a pool size of two. Subsequently, the data was flattened and passed through a dense output layer with a linear activation function. The number of units in the output layer corresponded to the number of targets (15 for both CNN models utilized for kinematics and kinetics prediction). The ‘Adam’ solver with a learning rate of 0.01 was used for weight optimization, employing the mean squared error as the loss function. An early stopping mechanism monitored validation loss and halted training if no improvement was observed after five epochs. The batch size was set to 32, and the model was trained for a maximum of 100 epochs to achieve robust results.

#### 2.4.5 Models’ evaluation

To evaluate the performance of the CNN and RF models, we began by extracting the super feature set from the windowed test dataset (Figure 2, Step 6). Subsequently, the trained ML models were employed to predict targets, joint kinematics, and kinetics using the extracted features from test datasets (Figure 2, Step 7). Then, we computed the root mean square error (RMSE) and Normalized RMSE (NRMSE) between the OpenSim outputs and the predicted values generated by each ML model for all targets. Violin plots were utilized to illustrate the distribution of RMSEs across various IMU configurations and examinations (intra and inter-subject) for each target. These plots offer a visual representation of how data is spread out within each category. In a violin plot, the width of the shape at any given point indicates the probability of values occurring. Additionally, within the violins, the median line is depicted as a short horizontal line, providing a clear reference point for the central tendency of the data. The reported RMSEs and NRMSEs for intra-subject and inter-subject examination are average of personalized and generalized models, respectively. After determining the optimal ML model and IMU sensor configuration, we conducted additional analysis by 1) plotting average waveforms from both OpenSim and ML models’ outputs, 2) calculating R2 values and creating correlation plots, and 3) generating Bland-Altman (Bland and Altman, 1986) plots to evaluate agreement between OpenSim outputs and predicted values for the selected model.

### 2.5 The effect of reducing IMU sensors to feet IMUs

In a prior study (Moghadam et al., 2023b), we demonstrated the feasibility of accurately estimating gait time series using machine learning models with just two IMUs positioned on the feet. To explore the applicability of this approach to children’s data, we replicated the steps described in Figure 2 using two IMUs placed on the feet instead of the full set of 7 IMUs.

## 3 Results

### 3.1 Joint kinematics prediction

**FIGURE 3:**
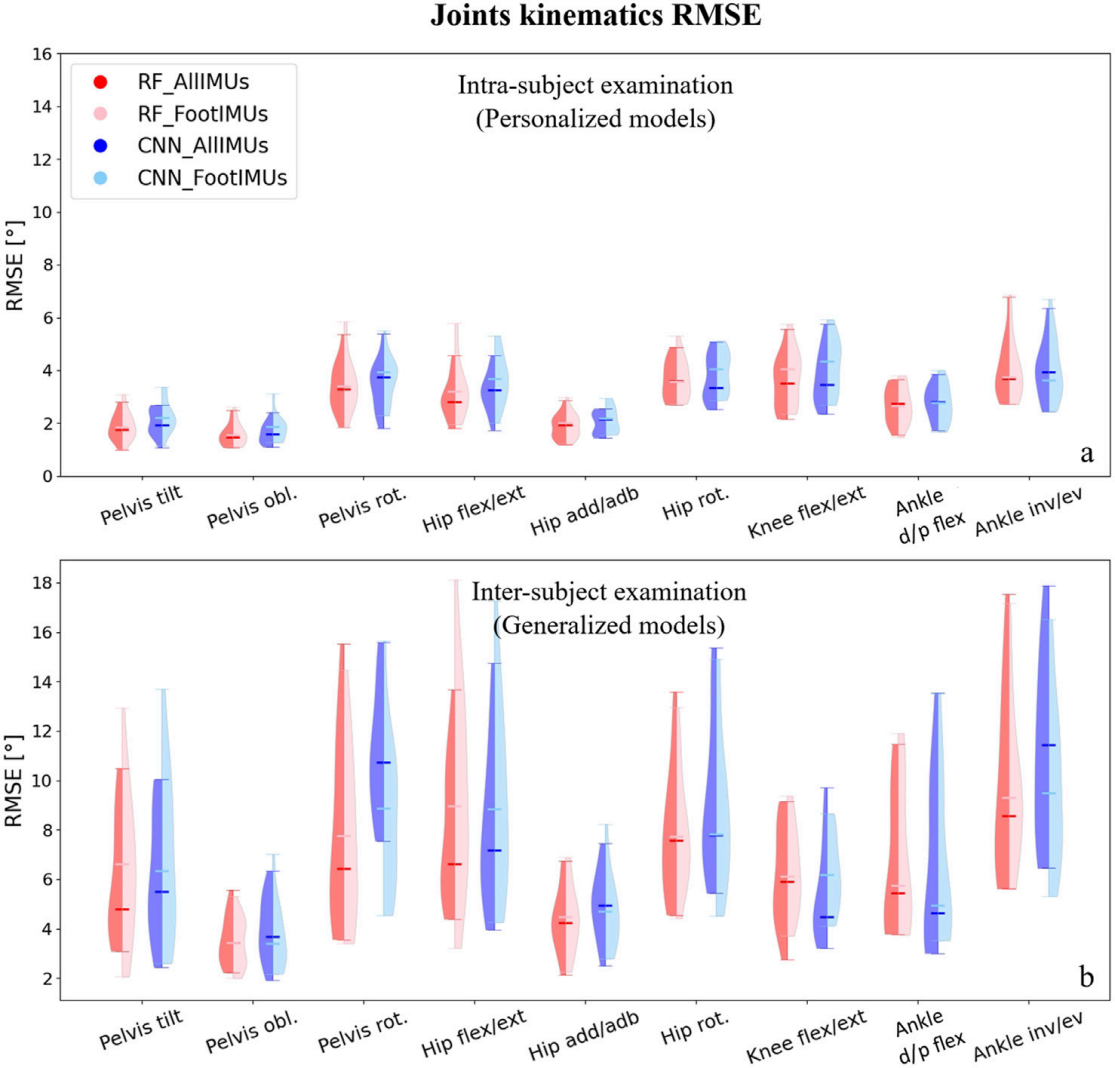
Violin plots illustrate the RMSE in degrees for joint kinematics predictions, comparing OpenSim IK outcomes with those from ML models. The red violins represent errors from the RF model, while the blue violins depict errors from the CNN model. Darker hues indicate models utilizing data from the full set of IMUs (*n* = 7), and lighter hues denote models using data solely from foot-mounted IMUs (*n* = 2). Panel **(A)** presents results from the intra-subject examination, while panel **(B)** displays the inter-subject examination results, utilizing models designed to generalize across participants.

The distribution of predicted joint kinematics RMSE revealed similar predictive accuracy between the RF and CNN models, whether we’re looking at the personalized models (intra-subject) or generalized models (inter-subject) (Figure 3). The RF model exhibited, on average, lower prediction errors of 0.22° in intra-subject examinations and 0.20° in inter-subject examinations when compared to the CNN model across all joints and planes of motion.

For the personalized models, reducing the number of IMUs to only one on each foot did not alter the prediction of joints kinematics compared to using all seven IMUs (Figure 3A). Interestingly, in the inter-subject examination, pelvis rotation, hip rotation, and ankle inversion/eversion angles experienced a decrease in their prediction errors by using only two IMUs; however, it increased the RMSE in the inter-subject evaluation for pelvis tilt and hip flexion/extension (Figure 3B). It is worth mentioning that these differences were not statistically significant.Independently of the model and the number of IMUs used, the average RMSE across all joints and planes of motion indicated considerably lower values in the intra-subject examinations compared to the inter-subject examinations. In the intra-subject evaluation, the RMSE spanned from a minimum of 1.0° (Pelvis tilt) to a maximum of 6.7° (ankle inversion/eversion). For the inter-subject evaluation, the range of RMSE increased, covering values from 2.1° (hip adduction/abduction) to 17.5° (ankle inversion/eversion).

In our analysis, we found that overall, the RF model gave slightly better results, and the number of IMUs used (two vs. seven) did not have an impact on the results. Therefore, we concentrated on the results provided by the RF model with two IMUs for the subsequent analysis. After normalizing the RMSE values to the data range, we observed that the lowest normalized RMSE (NRMSE) was associated with knee flexion/extension angle, and the highest NRMSE value was related to the pelvis tilt angle (Table 1). In the hip and ankle joint angles prediction, the lowest error appeared in the sagittal plane; however, in the case of the pelvis, the highest error was associated with the sagittal plane. This finding held true for both intra and inter-subject examinations. Notably, the NRMSE values for all joints and planes of motion in the inter-subject results were nearly twice as high as those observed in the intra-subject examination. Specifically, the average RMSE across all targets increased from 9.5% to 19.9% (Table 1). When comparing the average NRMSE for children below 10 years with children older than 10 years, a clear trend emerges. On average, the NRMSE is lower (1.7% in intra-subject and 0.3% in inter-subject examinations) in the older age group when predicting joint kinematics (refer to Supplementary Table SA1).

**TABLE 1:** The Normalised RMSE (NMRSE) along with their corresponding standard deviation (SD) values for joint angle prediction across all joints and planes of motion in intra and inter-subject examinations, based on RF models’ output using two IMUs.

	NRMSE (%) ± SD	
Joint kinematics target	Intra-subject examination	Inter-subject examination
Pelvis tilt	14.1 ± 5.1	33.1 ± 20.4
Pelvis obliquity	9.6 ± 2.7	19.7 ± 9.8
Pelvis rotation	13.8 ± 2.7	23.0 ± 13.1
Hip flexion/extension	6.1 ± 1.7	17.7 ± 8.4
Hip adduction/abduction	8.1 ± 1.9	18.4 ± 6.9
Hip rotation	11.9 ± 2.3	21.2 ± 7.1
Knee flexion/extension	5.2 ± 1.6	9.6 ± 6.6
Ankle dorsi/plantar flexion	6.7 ± 1.8	15.3 ± 5.7
Ankle inversion/eversion	10.1 ± 2.2	21.4 ± 5.9
Average	9.5 ± 3.3	19.9 ± 6.4

To understand if the prediction accuracy is consistent across the gait cycle for the intra-subject examination, we performed further analysis for the RF model outputs encompassing: 1) Average range of motion (ROM) comparison between the OpenSim IK tool and the RF model’s output. 2) Correlation plot and R-squared (*R* ^2^) Assessment, and 3) Bland-Altman Analysis to provide insights into the agreements between predicted and measured variables.

**FIGURE 4:**
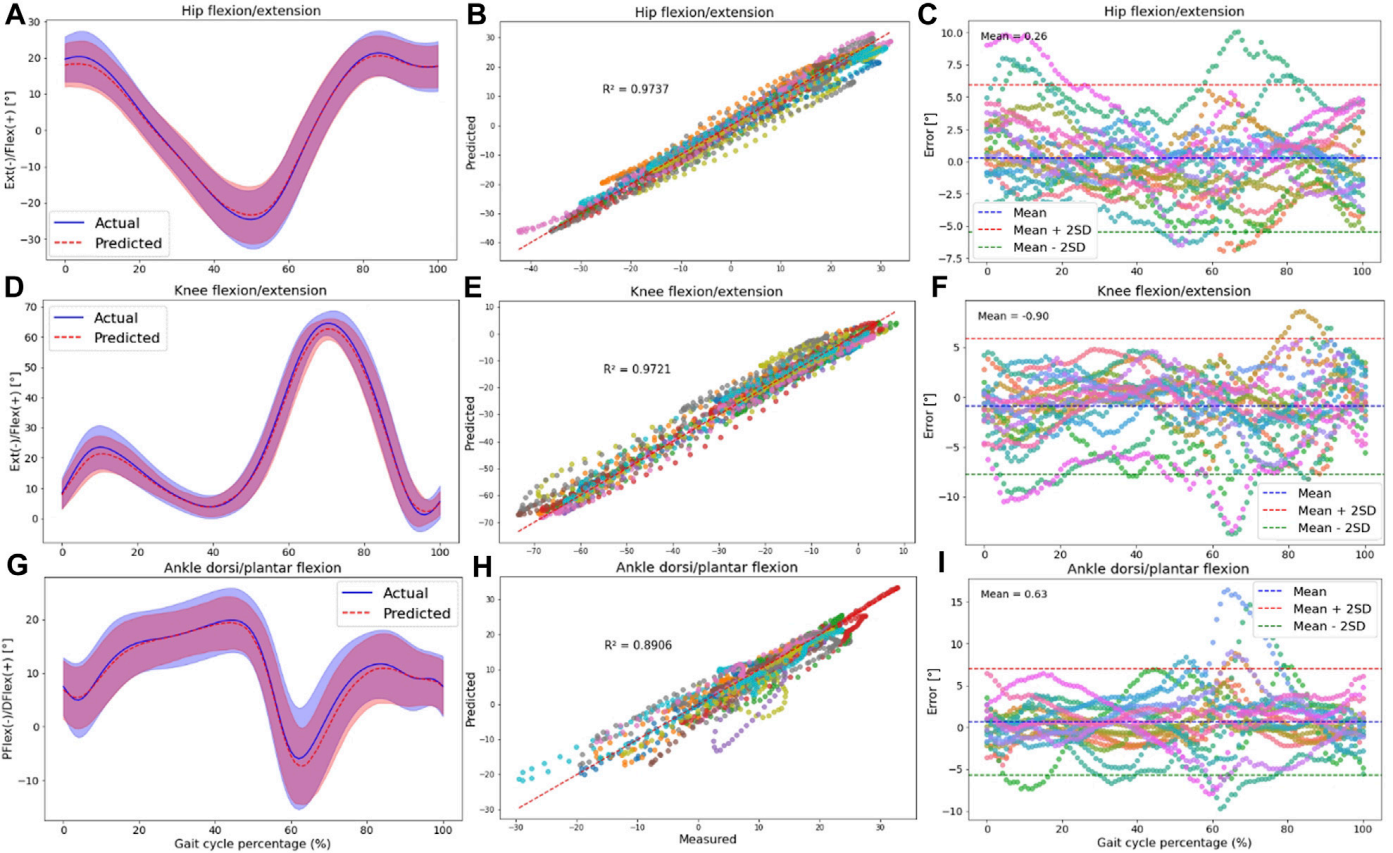
The plots are made across all participants in the intra-subject examination, specifically for hip **(A–C)**, knee **(D–F)**, and ankle **(G–I)** joint angles in the sagittal plane. Panels **(A,D,G)** present the RF model’s average predictions (the dashed red line represents the average, and the red shaded area indicates the SD) for joint angles, utilizing data from IMUs placed on the feet. These predictions are compared to the joint angles derived from the OpenSim IK tool (the solid blue line represents the average, and the blue shaded area indicates the SD). Panels **(B,E,H)** illustrate the correlation and R-squared (*R* ^2^) values for the mentioned joint angle targets. In **(C,F,I)**, we utilized Bland-Altman plots to visually depict the errors throughout one gait cycle for all participants. In these plots, the dashed blue line represents the mean error, and the mean ± 2SD is depicted as dashed red and green lines. Each distinct color in these plots represents the results of one participant.

Hip, knee, and ankle joint angles in the sagittal plane are presented in Figure 4. Additional results for other targets, including pelvis angles in all planes of motion, hip joint angles in the frontal and transverse planes, and ankle joint angles in the frontal plane, are detailed in Supplementary Figure SA1.

Plotting an average ROM (standard deviation (±SD)) across all participants revealed that the predicted waveforms closely followed the measured waveforms obtained from the OpenSim IK tool. The SD area of the predicted values fell within the shaded area representing the measured values, indicating a close fit between the predicted and measured data in the intra-subject examination (Figures 4A, D, G; Supplementary Figures SA1A, D, G, J, M, P).

Furthermore, there was a strong correlation between the OpenSim IK outputs and predicted joint angles, with *R* ^2^ values exceeding 0.83 for pelvis angles in all planes of motion (Supplementary Figures SA1B, E, H), 0.76 for hip angles (Supplementary Figures SA1K, N; Figure 4B), 0.97 for knee angle (Figure 4E), and 0.77 for ankle joint angles (Supplementary Figures SA1Q; Figure 4H). The high performance of the RF model in the sagittal plane was observed at the hip, knee, and ankle.

Strong agreement between the measured and predicted values was evident in the Bland-Altman plots, with the error falling within the range of two standard deviations from the mean value for most participants. No specific pattern in the error values was seen based on these plots; however, the bias between predicted and measured kinematics was around zero for all targets (Figures 4C, F, I; Supplementary Figures SA1C, F, I, L, O, R).

### 3.2 Joint kinetics prediction

**FIGURE 5:**
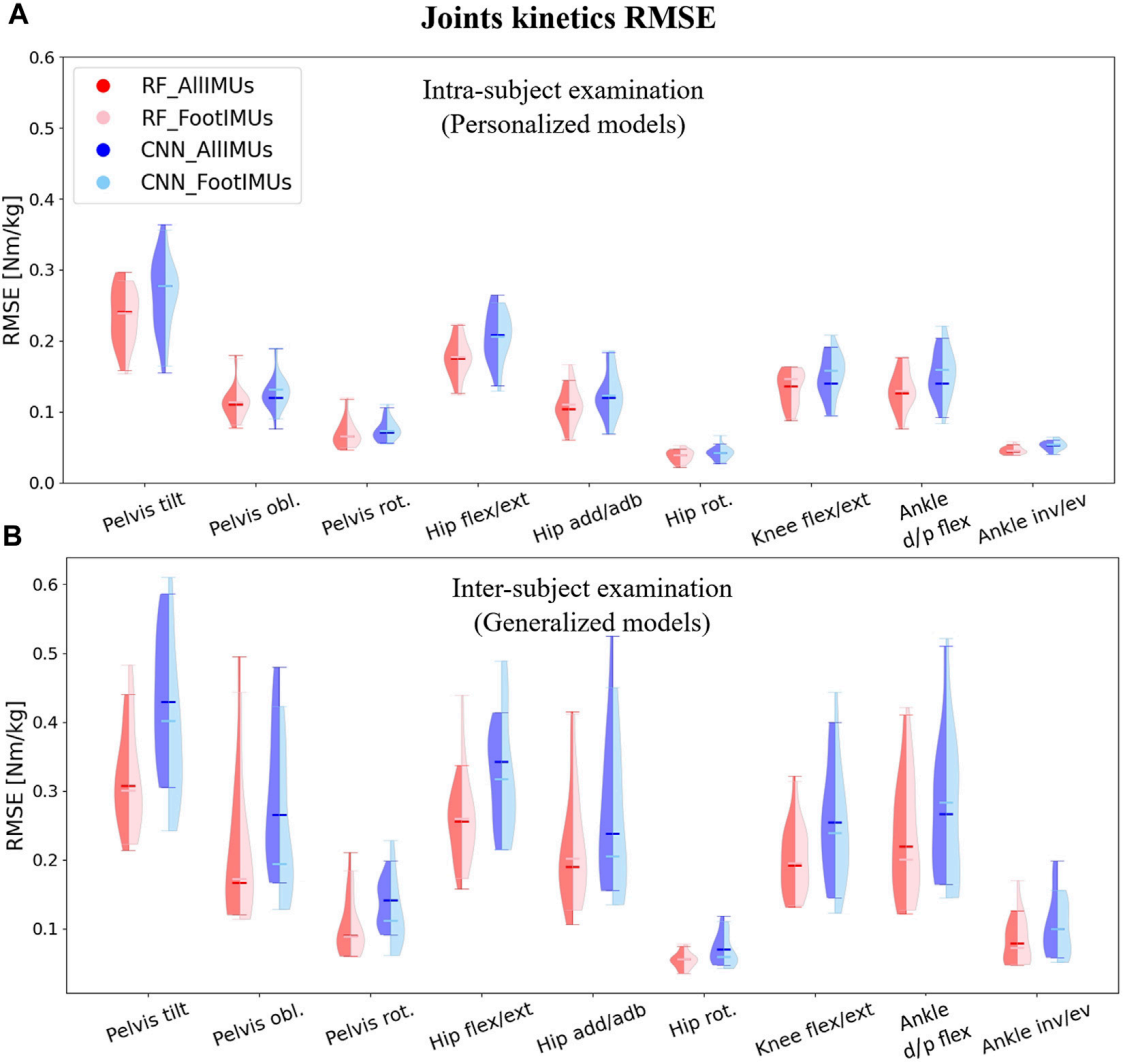
Violin plots illustrate the RMSE in degrees for joint kinematics predictions, comparing OpenSim ID outcomes with those from ML models. The red violins represent errors from the RF model, while the blue violins depict errors from the CNN model. Darker hues indicate models utilizing data from the full set of IMUs (*n* = 7), and lighter hues denote models using data solely from foot-mounted IMUs (*n* = 2). Panel **(A)** presents results from the intra-subject examination, while panel **(B)** displays the inter-subject examination results, utilizing models designed to generalize across participants.

When predicting joint kinetics, the RF model demonstrated slightly superior performance when compared to the CNN model (Figure 5). The RF model yielded lower prediction error values than the CNN model across all joints and planes of motion, with a reduction of 0.017 Nm/kg RMSE in intra-subject examinations and 0.037 Nm/kg RMSE in inter-subject examinations. The better performance of the RF model was more pronounced in the inter-subject examination (Figure 5B).

In terms of the number of IMU sensors used for joint kinetics prediction, we found nearly identical results when employing only the feet IMUs as compared to using all 7 IMUs. However, in specific kinetics targets, such as pelvis tilt and hip flexion/extension in the intra-subject examination and pelvis obliquity, hip adduction/abduction, and hip rotation in the inter-subject examination, even lower prediction errors were achieved by utilizing just two IMUs placed on the feet. It is important to highlight that irrespective of the model type and the number of IMUs employed for prediction, the RMSE values in the inter-subject examination were consistently higher than the RMSE in the intra-subject examination.

As for the kinematics, we concentrated on the results provided by the RF model with two IMUs for further analysis. After calculating the NRMSE between outputs of the RF model and the OpenSim ID tool, we observed that the highest NRMSE values were associated with the pelvis tilt in the intra-subject examination (similar to the kinematics analysis) and hip flexion/extension in the inter-subject examination. Conversely, ankle dorsi/plantar flexion exhibited the lowest NRMSE for intra-subject examination, while knee flexion/extension displayed the lowest NRMSE for inter-subject examination.

Just like with joint kinematics, the NRMSE values for all joints and planes of motion in the inter-subject results were higher than the intra-subject examination. Specifically, the average RMSE across all targets increased from 10.7% to 15.2% (Table 2). Similar to the prediction of joint kinematics, a consistent trend was noted in the prediction of joint kinetics (refer to Supplementary Table SA2). Notably, the NRMSE was lower by 1% in intra-subject and 4.3% in inter-subject examinations for older children (above 10 years old) as opposed to their younger counterparts (below 10 years old).

**TABLE 2:** the NRMSE values along with their corresponding SD for joint moment prediction across all joints and planes of motion in intra and inter-subject examinations, based on RF models’ output using feet IMUs.

	NRMSE (%) ± SD	
Joint kinetics target	Intra-subject examination	Inter-subject examination
Pelvis tilt	13.7 ± 1.9	13.9 ± 3.6
Pelvis obliquity	13.2 ± 2.1	16.8 ± 5.2
Pelvis rotation	12.8 ± 3.4	13.4 ± 3.8
Hip flexion/extension	11.8 ± 2.1	26.4 ± 14.5
Hip adduction/abduction	8.5 ± 2.1	15.1 ± 8.8
Hip rotation	9.6 ± 2.9	10.9 ± 3.3
Knee flexion/extension	8.5 ± 1.9	10.3 ± 2.6
Ankle dorsi/plantar flexion	6.4 ± 1.3	11.7 ± 9.6
Ankle inversion/eversion	12.5 ± 2.3	18.5 ± 7.3
Average	10.7 ± 2.6	15.2 ± 4.9

To understand if the prediction accuracy is consistent across the gait cycle for the intra-subject examination, we performed further analysis for the RF model outputs encompassing: 1) Average normalized moment comparison between the OpenSim IK tool and the RF model’s output. 2) Correlation plot and R-squared (*R* ^2^) Assessment, and 3) Bland-Altman Analysis to provide insights into the agreements between predicted and measured variables.

**FIGURE 6:**
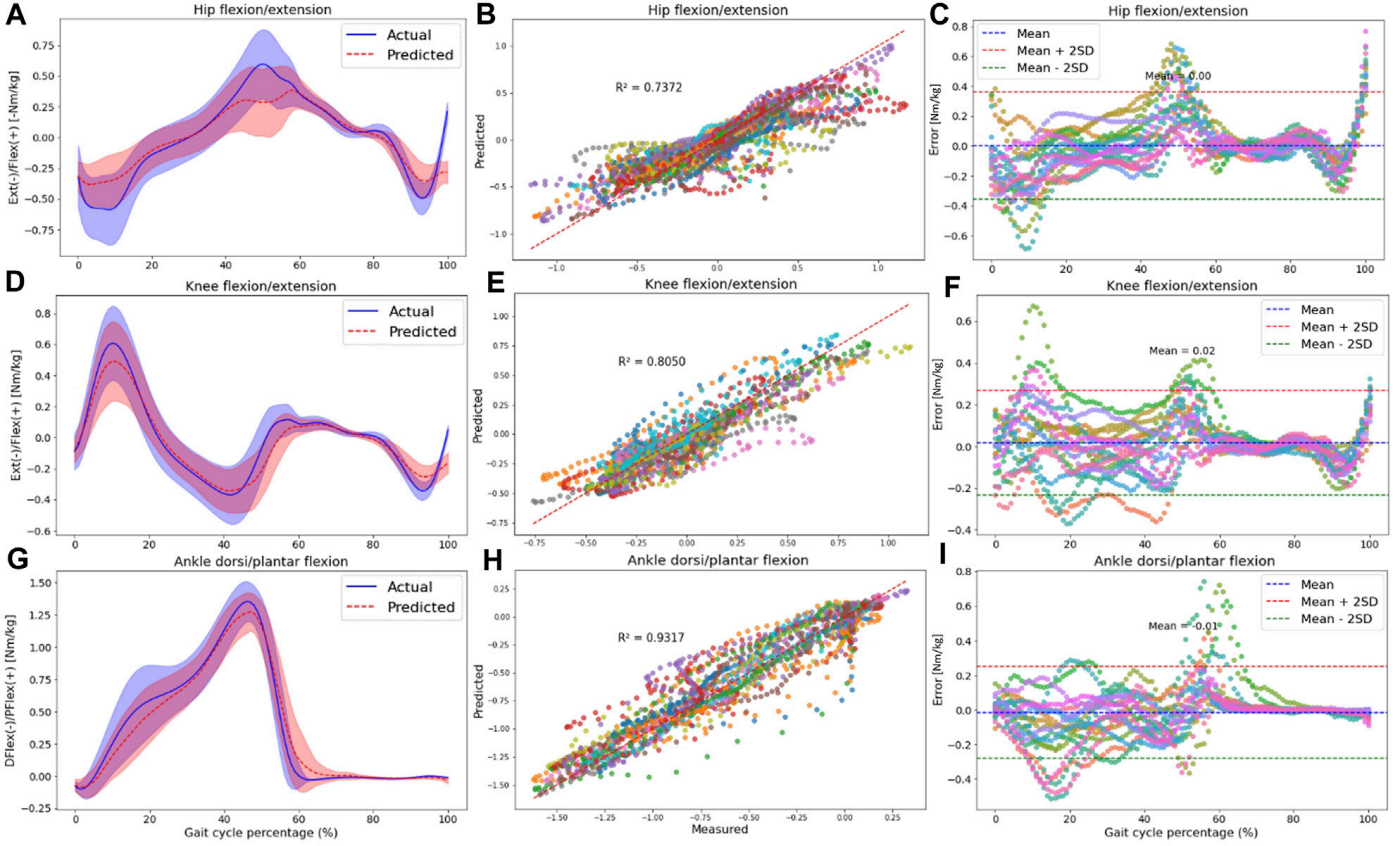
The plots are made across all participants in the intra-subject examination, specifically for hip **(A–C)**, knee **(D–F)**, and ankle **(G–I)** joint moments in the sagittal plane. Panels **(A,D,G)** present the RF model’s average predictions (the dashed red line represents the average, and the red shaded area indicates one SD) for joint moments, utilizing data from IMUs placed on the feet. These predictions are compared to the joint moments derived from the OpenSim ID tool (the solid blue line represents the average, and the blue shaded area indicates one SD). Panels **(B,E,H)** illustrate the correlation and R-squared (*R* ^2^) values for the mentioned joint moment targets. In **(C,F,I)**, we utilized Bland-Altman plots to visually depict the errors throughout one gait cycle for all participants. In these plots, the dashed blue line represents the mean error, and the mean ± 2SD is depicted as dashed red and green lines. Each distinct color in correlation and error plots represents the results of one participant.

The results for the hip, knee, and ankle joint moments in the sagittal plane are shown in Figure 6. Additional results for other targets, including pelvis moments in all planes of motion, hip joint moments in the frontal and transverse planes, and ankle joint moment in the frontal plane, are detailed in Supplementary Figure SA2.

Plotting the average and standard deviation waveforms for joint moments throughout a gait cycle in intra-subject examination, we observed that the RF model’s predictions effectively tracked the OpenSim ID tool output. However, the SD area of the predicted values did not consistently fall within the shaded area related to the measured values across the entire gait cycle [(Figures 6A, D, G); Supplementary Figures SA2A, D, G, J, M, P]. Especially toward the end of the stance phase (50%–60% of the gait cycle), the model predicted a lower range of motion in the three joints in the sagittal plane. Also, at the beginning of the stance phase, we can observe some discrepancies in knee flexion and hip extension moment predictions.

Compared to joint angles, the correlation between the measured and predicted joint moments was lower for the pelvis, with R2 values of 0.43, 0.31, and 0.41 for pelvis tilt, obliquity, and rotation, respectively (Supplementary Figures SA2B, E, H). The R2 values for other joint kinetics were consistent with the performance seen in joint kinematics prediction. Specifically, the hip joint experienced R2 higher than 0.73 in all planes of motion (Supplementary Figures SA2K, N; Figure 6B), the R2 for the knee joint was 0.80 in the sagittal plane (Figure 6E), and the ankle displayed R2 values higher than 0.83 in both the frontal (Supplementary Figures SA2Q) and sagittal planes (Figure 6H).

According to Bland-Altman plots, there was a good agreement between the measured and predicted targets, as the errors were within a range of two SD of the mean value for most participants. Similar to joint kinematics prediction, the bias between measured and predicted variables was around zero for all kinetics targets. An interesting observation was that during the final phase of the gait cycle (swing phase), errors were almost zero for most joint kinetics (Figures 6C, F, I; Supplementary Figures SA1C, F, I, L, O, R).

## 4 Discussion

The aim of this study was to investigate the feasibility of using a combination of IMUs’ data and ML models for predicting joint kinematics and kinetics in school-aged children. To answer this aim, the first objective was to assess the accuracy of RF and CNN ML models by quantifying both intra-subject and inter-subject prediction errors. The second objective was to evaluate the influence of using only an IMU on each foot versus seven IMUs, one on each segment of the lower limb, on kinematics and kinetics prediction performance. We employed a feature engineering technique to extract and select the most important features from the IMUs’ acceleration and angular velocity data to enhance the models’ performance.

### 4.1 ML comparison

Regarding the first objective, findings suggested that the RF and CNN models demonstrated comparable performance for predicting joint kinematics (NRMSE of 9.5% versus 10.6% for personalized and NRMSE of 19.9% versus 22.5% for generalized models, respectively) and kinetics (NRMSE of 10.7% versus 12.9% for personalized and NRMSE of 15.2% versus 17.9% for generalized models respectively) in TD children. This implies that the complexity of deep neural network structures may not be necessary for gait time series prediction. Consequently, it opens the door to more efficient and easily interpretable modeling approaches, such as the RF model (Breiman, 2001). Supporting this notion, a separate study found RF models to outperform CNN models in estimating step length, showing an absolute error of 5.09 cm for RF compared to 5.26 cm for CNN (Seifer et al., 2023). Furthermore, the superiority of RF models, with an average error of 5.57°, becomes evident in gait trajectory generation, surpassing the neural network model with an average error of 6.00° in another study (Ren et al., 2019).

The higher performance of RF models could be attributed to their resilience against overfitting. This resilience arises from their capacity to amalgamate multiple decision trees trained on bootstrapped data, coupled with the utilization of feature randomization, pruning, and averaging (Breiman, 2001). On the other hand, CNNs exhibit a notable susceptibility to overfitting, particularly when dealing with smaller datasets (Slijepcevic et al., 2023).

#### 4.1.1 Intra-subject examination

We created 17 personalized models for predicting kinematics and kinetics based on customized feature sets specific to each participant. Our results from the RF model using two IMUs data demonstrated strong predictive accuracy, with an average RMSE ranging from 1.61° to 4.16° (NRMSE of 5.2%–14.1%) across all joint kinematics. The RMSE values for joint kinematics stayed well below the 5° error threshold, which is often considered a clinically acceptable level of deviation for assessing joint movements (Slater et al., 2018). However, the joint kinematics prediction error were higher than for adults in other studies, where observed values ranged from 1.38° to 3.96° for all targets (Findlow et al., 2008; Giarmatzis et al., 2020; Moghadam et al., 2023a; Yeung et al., 2023). Similarly, joint kinetics prediction error were higher (0.038–0.233 Nm/kg) in this study than on adult population, where the RMSE ranged from 0.042 to 0.198 Nm/kg (Dey et al., 2019; Mundt et al., 2020; Moghadam et al., 2023a). This elevated error in both kinematics and kinetics prediction in the intra-subject evaluation of children compared to the adult models underscores the greater variability in gait time series within individual children across different trials. Different gait maturity level (Bach et al., 2021) as well as heightened variability in gait patterns (Kuhtz-Buschbeck et al., 1996) and EMG gait waveforms (Granata et al., 2005) in children compared to adults has been shown in other studies. This discrepancy may be attributed to the increased susceptibility of children to distractions during walking (Stolze et al., 1998) or to the heterogeneity in children’s gait cycles compared to adults.

Despite the higher errors in the children’s personalized ML model compared to adults, the Bland-Altman plots revealed a notable level of agreement between the measured and predicted values during the intra-subject examination. Most participants exhibited errors within the range of two SD from the mean error value. Additionally, average errors consistently remained near zero for all predicted targets, underlining the good overall agreement between the IMU-based and OMC-based kinematics and kinetics. It is worth noting that no discernible patterns in the error values were observed, indicating a lack of systematic bias in the predictions (Bland and Altman, 1986). These findings emphasize the practicality and suitability of employing this approach, which involves a personalized RF model utilizing IMU data for accurately estimating gait time series in children.

One interesting finding of personalized modeling (intra-subject examination) was the good model performance within the sagittal plane compared to the other planes of motion for joint kinematics prediction, especially in the case of hip and ankle joint angles. The knee angle, only computed within the sagittal plane, demonstrated a high correlation between the actual and predicted values (R2 of 0.97). The enhanced performance of the RF model in the sagittal plane (higher R2 and lower errors) can be attributed to the more prominent joint movements within this plane, which, in turn, yields more distinct signals from the IMUs. Consequently, this clarity in the IMU signals contributes to the model’s improved predictive performance in the sagittal plane. While other planes of motion, such as the frontal and transverse planes, contribute to a comprehensive understanding of gait, the sagittal plane takes precedence in gait analysis due to its primary role in capturing the fundamental aspects of forward movement. We’ve shown that the proposed personalized method exhibits remarkable accuracy, demonstrating a clinically acceptable level of error, particularly in the sagittal plane. This notable precision positions it as exceptionally valuable for advancing the gait analysis of children.

#### 4.1.2 Inter-subject examination

In the inter-subject examination of the RF model, the results were less promising compared to the intra-subject test, with average RMSE ranging from 3.5° to 9.6° (NRMSE of 9.6%–33.1%) for joint kinematics. When comparing the outcomes with adults cohort, it becomes apparent that the RMSE values in adults exhibit lower errors (RMSE between 2.17° and 6.53°) (Luu et al., 2014; Dorschky et al., 2020; Lim et al., 2020; Sharifi Renani et al., 2021; Moghadam et al., 2023a). Similar findings were found for joint moments with NRMSE of 10.3%–26.4% found in this study compared to 4.54%–10.74% in previous adults studies (Giarmatzis et al., 2020; Lim et al., 2020). In fact, these errors are of such magnitude that they do not provide confidence in the accurate prediction of gait time series in children not included in the training set. This contrasts with the previously demonstrated success of inter-subject modeling in predicting time series for the adult population with limited data (Giarmatzis et al., 2020; Lim et al., 2020; Stetter et al., 2020; Moghadam et al., 2023a).

The primary reason for the elevated error in the children’s generalized model compared to adults can be attributed to the diverse gait patterns among individual children, given their ongoing musculoskeletal changes and developmental stages within the specified age range of six to 15 years in this study (Onis et al., 2007; Bari et al., 2023). However, as age advances, there is a reduction in variability within the gait pattern, as demonstrated in our findings revealing higher errors in gait analysis for younger children compared to older ones. When analysing the average (±SD) waveforms of targets within a gait cycle, we observed a noticeable standard deviation surrounding the average waveform for the children, reaffirming the high variability of gait patterns among children. This is comparable to a study by Fokuchi et al., where a greater deviation area for younger people compared to the adults’ normative gait data is shown (Fukuchi et al., 2018). The secondary reason for the high error in generalized modeling lies in the limitations of the dataset. Effective ML models typically require access to extensive datasets comprising a wide spectrum of walking patterns. Consequently, the performance of a model trained on a small dataset featuring only 16 participants is inherently limited when applied to new, unseen subjects. The considerable errors observed in generalized modeling render this approach less advisable for children who were not part of the initial training dataset.

### 4.2 Number of IMUs

Concerning the second objective, we demonstrated that utilizing two IMUs on the feet, instead of a total of seven IMUs, resulted in similar accuracy of the models, specifically in intra-subject examination. Concerning the inter-subject examination, while there was a slight increase in error for some targets (pelvis tilt, hip flexion/extension angles and moments, and ankle dorsi/plantar moment), reducing the number of IMUs to feet IMUs resulted in decreased errors for specific targets such as pelvis rotation angle, hip rotation angle, ankle inversion/eversion angles, pelvis obliquity moment, hip adduction/abduction moment, and hip rotation moment.

These findings are consistent with our prior research, which suggested that in adult gait prediction, employing ML models allows us to achieve nearly identical results using only feet IMUs, as opposed to utilizing seven IMUs (one for each segment) (Moghadam et al., 2023b). This can be attributed to the proficiency of ML models in establishing a robust relationship between IMU data and targets. Another contributing factor is the identification of alternative features to raw IMU data, thereby augmenting the predictive capabilities of the ML model, even when working with a limited number of IMUs. The feasibility of employing a single IMU on the pelvis (Lim et al., 2020) or a pair of IMUs on shanks (Sharifi Renani et al., 2020; Yeung et al., 2023) or feet (Gholami et al., 2020) for predicting a diverse range of gait time series has been demonstrated in prior studies. Reducing the number of IMUs streamlines model implementation, decreases data processing time, and lowers sensor-related costs. Additionally, the potential integration of foot IMUs within shoes, rendering them inconspicuous during community use, could enhance patient compliance.

### 4.3 Limitations and future work

This study presents limitations to be addressed by future research. Firstly, the utilization of a generic adult model for scaling and constructing musculoskeletal models for children. The issue lies in the potential discrepancies between generic adult models and the individual anatomical characteristics of children. Developing more precise, subject-specific models would ideally involve leveraging medical imaging data, such as MRI, CT, or X-rays, which can be both time-consuming and financially burdensome (Nolte et al., 2016). To address this limitation, future research could explore alternative methods like statistical shape modeling to build children’s musculoskeletal models (Carman et al., 2022).

Another limitation of this study pertains to the omission of an investigation into the potential impact of slight variations in the placement of IMUs that may occur when different individuals are responsible for placing the IMUs. The concern here is that small variations could influence the data collected and, consequently, affect the accuracy and reliability of the results. Addressing this limitation in future research might involve conducting a sensitivity analysis or implementing standardized procedures for IMU placement to mitigate the potential impact of such variations on the study outcomes. It is noteworthy that a similar analysis was taken in an adult study and did not change the results (Moghadam et al., 2023a). So, we expect that these findings apply to children, too, meaning small changes in sensors’ placement should not substantially affect the outcomes.

Our study focused on TD children to establish the models, which will differ from other populations, such as children with cerebral palsy. The choice of ML model and the number of required IMUs may differ, as children with movement disorders often exhibit more complex and diverse gait patterns. Several research groups have successfully employed regression machine learning models to estimate gait time series in specific patient cohorts. Examples of previous studies on patients include the prediction of knee joint moments during gait in individuals with CP (Kwon et al., 2012), the estimation of knee joint kinematics in patients with knee osteoarthritis (Tan et al., 2022), and the forecasting of gait parameters for patients with osteoarthritis (OA) and those undergoing total knee arthroplasty (TKA) (Sharifi Renani et al., 2020). Notably, these investigations demonstrated high correlation coefficients ranging from 0.71 to 0.99, showcasing the viability of gait time series prediction in targeted patient groups using wearable sensors and machine learning models. While the model employed in this study demonstrates robust performance with TD children, its suitability for diverse pathologies warrants exploration. Gait patterns vary significantly across different conditions, making it imprudent to apply the exact same model to a new population. Consequently, our future endeavours will involve evaluating the performance of our algorithm on additional patient cohorts, including children with cerebral palsy.

Another notable limitation is the computational resources required for the primary feature extraction and selection processes. We utilized high-performance computers with 80 GB of RAM memory to address this demand. However, once the model is trained, it can be executed on less powerful computers, focusing solely on extracting the selected features and providing inference from the model.

It is also important to acknowledge that the accuracy of estimations using data from other labs may not match the precision of our own results. This discrepancy can be related to variations in equipment and sensor usage across different laboratories. However, by incorporating data from multiple labs into the training dataset for our models, we can enhance the models’ ability to generalize across different settings. In future work, it would also be valuable to consider the integration of a contactless monitoring system, akin to the innovative approach developed in a separate study (Huang et al., 2024). Integrating such systems into the ML model holds the potential for real-time prediction of gait time series in children.

### 4.4 Strengths and contributions

By developing an ML model for predicting gait time series in children with diverse gait patterns, we achieved results comparable to studies focused on the adult population, particularly in the context of personalized modeling. We believe that our model offers several advantages over traditional methods that rely on IMUs for gait analysis. For instance, our model can predict a comprehensive set of lower limb joint angles and moments during gait using only two IMUs attached to the feet. To the best of our knowledge, this is the first study tailored to children’s gait time series prediction, leveraging a combination of IMU data and ML techniques.

Other methodologies, which would use sensor fusion algorithms rather than ML, require additional normalization steps to calculate each IMU sensors’ orientation relative orientation to each body segment, leading to inaccuracies and numerical drift errors. In contrast, our personalized models have good accuracy, can be streamlined, and work independently of the user’s expertise. Following a single data collection session in a gait lab, remote patient monitoring becomes feasible by placing IMUs on the patient’s feet and feeding the IMU data into the model for inference. Furthermore, this workflow can be utilized in real-time, as the inference time for the RF model is on the order of milliseconds.

## 5 Conclusion

The current study showed that RF and CNN models exhibit comparable results in the context of gait analysis within a typically developed pediatric population. The practicality of employing only two IMUs placed on the feet for predicting a comprehensive set of lower-limb joint kinematics and kinetics was successfully demonstrated. The presented workflow, employing foot IMUs, not only reduces processing time but also streamlines the integration of wearable sensors in clinical settings. Our forthcoming research endeavors will include increasing the sample size and introducing more variability to the overground walking scenarios to enhance the accuracy of our generalized model. Moreover, future work will be dedicated to developing ML models tailored to a cohort of children with movement disorders, specifically children with CP. This expansion promises to bring valuable insights and tools to the field of pediatric gait analysis, serving as a testament to the potential for advanced technology to benefit those with unique clinical requirements.

## Data Availability

The datasets presented in this study can be found in online repositories. The names of the repository/repositories and accession number(s) can be found below: The post-processed data, including joint kinematics, joint kinetics, as well as the raw IMU data utilized for constructing ML models in this study, are accessible on the open-source platform SimTK.org (https://simtk.org/projects/ml_sensors).

## References

[B1] Al Borno M. O’Day J. Ibarra V. Dunne J. Seth A. Habib A. (2022). Opensense: an open-source toolbox for inertial-measurement-unit-based measurement of lower extremity kinematics over long durations. J. neuroengineering rehabilitation 19 (1), 22–11. 10.1186/s12984-022-01001-x PMC8859896 35184727

[B2] Aminian K. Najafi B. (2004). Capturing human motion using body‐fixed sensors: outdoor measurement and clinical applications. Comput. Animat. virtual worlds 15 (2), 79–94. 10.1002/cav.2

[B3] Bach M. M. Daffertshofer A. Dominici N. (2021). The development of mature gait patterns in children during walking and running. Eur. J. Appl. physiology 121, 1073–1085. 10.1007/s00421-020-04592-2 PMC7966230 33439307

[B4] Bakke D. Besier T. (2022). Shape-model scaled gait models can neglect segment markers without consequential change to inverse kinematics results. J. Biomechanics 137, 111086. 10.1016/j.jbiomech.2022.111086 35436755

[B5] Bari M. A. Mir H. N. Parrey J. A. Ateeq A. Ajhar A. Al Muslem W. H. (2023). Exploring variations in gait patterns and joint motion characteristics in school-aged children across different walking speeds: a comprehensive motion analysis study. J. Med. Life 16 (6), 895–903. 10.25122/jml-2023-0110 37675178 PMC10478655

[B6] Benjamini Y. Hochberg Y. (1995). Controlling the false discovery rate: a practical and powerful approach to multiple testing. J. R. Stat. Soc. Ser. B Methodol. 57 (1), 289–300. 10.1111/j.2517-6161.1995.tb02031.x

[B7] Bland J. M. Altman D. (1986). Statistical methods for assessing agreement between two methods of clinical measurement. lancet 327 (8476), 307–310. 10.1016/s0140-6736(86)90837-8 2868172

[B8] Breiman L. (2001). Random forests. Mach. Learn. 45 (1), 5–32. 10.1023/a:1010933404324

[B9] Carman L. Besier T. F. Choisne J. (2022). Morphological variation in paediatric lower limb bones. Sci. Rep. 12 (1), 3251. 10.1038/s41598-022-07267-4 35228607 PMC8885755

[B10] Chester V. L. Biden E. N. Tingley M. (2005). Gait analysis. Biomed. Instrum. Technol. 39 (1), 64–74. 10.2345/0899-8205(2005)39[64:GA]2.0.CO;2 15742852

[B11] Choisne J. Fourrier N. Handsfield G. Signal N. Taylor D. Wilson N. (2020). An unsupervised data-driven model to classify gait patterns in children with cerebral palsy. J. Clin. Med. 9 (5), 1432. 10.3390/jcm9051432 32408489 PMC7290444

[B12] Christ M. Braun N. Neuffer J. Kempa-Liehr A. W. (2018). Time series feature extraction on basis of scalable Hypothesis tests (Tsfresh–a Python package). Neurocomputing 307, 72–77. 10.1016/j.neucom.2018.03.067

[B13] Cigali B. S. Uluçam E. Bozer C. (2011). 3d motion analysis of hip, knee and ankle joints of children aged between 7-11 Years during gait. Balkan Med. J. (2), 197–201. 10.5174/tutfd.2010.04199.2

[B14] Davis R. B. III Ounpuu S. Tyburski D. Gage J. R. (1991). A gait analysis data collection and reduction technique. Hum. Mov. Sci. 10 (5), 575–587. 10.1016/0167-9457(91)90046-z

[B15] Delp S. L. Anderson F. C. Arnold A. S. Loan P. Habib A. John C. T. (2007). Opensim: open-source software to create and analyze dynamic simulations of movement. IEEE Trans. Biomed. Eng. 54 (11), 1940–1950. 10.1109/tbme.2007.901024 18018689

[B16] Dey S. Yoshida T. Ernst M. Schmalz T. Schilling A. F. (2019). “A random forest approach for continuous prediction of joint angles and moments during walking: an implication for controlling active knee-ankle prostheses/orthoses,” in 2019 IEEE International Conference on Cyborg and Bionic Systems (CBS) (IEEE), 66–71. 10.1109/ICORR.2019.8779445 31374717

[B17] Dorschky E. Nitschke M. Martindale C. F. Van den Bogert A. J. Koelewijn A. D. Eskofier B. M. (2020). Cnn-based estimation of sagittal plane walking and running Biomechanics from measured and simulated inertial sensor data. Front. Bioeng. Biotechnol. 8, 604. 10.3389/fbioe.2020.00604 32671032 PMC7333079

[B18] Findlow A. Goulermas J. Nester C. Howard D. Kenney L. (2008). Predicting lower limb joint kinematics using wearable motion sensors. Gait posture 28 (1), 120–126. 10.1016/j.gaitpost.2007.11.001 18093834

[B19] Fukuchi C. A. Fukuchi R. K. Duarte M. (2018). A public dataset of overground and treadmill walking kinematics and kinetics in healthy individuals. PeerJ 6, e4640. 10.7717/peerj.4640 29707431 PMC5922232

[B20] Ganley K. J. Powers C. M. (2005). Gait kinematics and kinetics of 7-year-old children: a comparison to adults using age-specific anthropometric data. Gait posture 21 (2), 141–145. 10.1016/j.gaitpost.2004.01.007 15639392

[B21] Gholami M. Napier C. Menon C. (2020). Estimating lower extremity running gait kinematics with a single accelerometer: a deep learning approach. Sensors 20 (10), 2939. 10.3390/s20102939 32455927 PMC7287664

[B22] Giarmatzis G. Zacharaki E. I. Moustakas K. (2020). Real-time prediction of joint forces by motion capture and machine learning. Sensors 20 (23), 6933. 10.3390/s20236933 33291594 PMC7730598

[B23] Granata K. P. Padua D. A. Abel M. F. (2005). Repeatability of surface emg during gait in children. Gait Posture 22 (4), 346–350. 10.1016/j.gaitpost.2004.11.014 16274917 PMC1628350

[B24] Gurchiek R. D. Cheney N. McGinnis R. S. (2019). Estimating biomechanical time-series with wearable sensors: a systematic review of machine learning techniques. Sensors 19 (23), 5227. 10.3390/s19235227 31795151 PMC6928851

[B25] Harrington M. Zavatsky A. Lawson S. Yuan Z. Theologis T. (2007). Prediction of the hip joint centre in adults, children, and patients with cerebral palsy based on magnetic resonance imaging. J. biomechanics 40 (3), 595–602. 10.1016/j.jbiomech.2006.02.003 16584737

[B26] Hasan M. A. M. Nasser M. Ahmad S. Molla K. I. (2016). Feature selection for intrusion detection using random forest. J. Inf. Secur. 7 (3), 129–140. 10.4236/jis.2016.73009

[B27] Huang S. Dai H. Yu X. Wu X. Wang K. Hu J. (2024). A contactless monitoring system for accurately predicting energy expenditure during treadmill walking based on an ensemble neural network. Iscience 27 (3), 109093. 10.1016/j.isci.2024.109093 38375238 PMC10875158

[B28] Ito T. Noritake K. Ito Y. Tomita H. Mizusawa J. Sugiura H. (2022). Three-dimensional gait analysis of lower extremity gait parameters in Japanese children aged 6 to 12 years. Sci. Rep. 12 (1), 7822. 10.1038/s41598-022-11906-1 35551257 PMC9098504

[B29] Jain E. Anthony L. Aloba A. Castonguay A. Cuba I. Shaw A. (2016). Is the motion of a child perceivably different from the motion of an adult? ACM Trans. Appl. Percept. (TAP) 13 (4), 1–17. 10.1145/2947616

[B30] Kamruzzaman J. Begg R. K. (2006). Support vector machines and other pattern recognition approaches to the diagnosis of cerebral palsy gait. IEEE Trans. Biomed. Eng. 53 (12), 2479–2490. 10.1109/tbme.2006.883697 17153205

[B31] Khaksar S. Pan H. Borazjani B. Murray I. Agrawal H. Liu W. (2021). Application of inertial measurement units and machine learning classification in cerebral palsy: randomized controlled trial. JMIR Rehabilitation Assistive Technol. 8 (4), e29769. 10.2196/29769 PMC8567153 34668870

[B32] Knudson D. V. (2007). Fundamentals of Biomechanics. Springer.

[B33] Kolaghassi R. Al-Hares M. K. Marcelli G. Sirlantzis K. (2022). Performance of deep learning models in forecasting gait trajectories of children with neurological disorders. Sensors 22 (8), 2969. 10.3390/s22082969 35458954 PMC9033153

[B34] Kolaghassi R. Marcelli G. Sirlantzis K. (2023). Deep learning models for stable gait prediction applied to exoskeleton reference trajectories for children with cerebral palsy. IEEE Access 11, 31962–31976. 10.1109/access.2023.3252916

[B35] Kuhtz-Buschbeck J. Boczek-Funcke A. Heinrichs H. Illert M. Stolze H. (1996). Kinematic analysis of prehension in children. Eur. J. Neurosci. Suppl, 9–131. 10.1016/s0166-4328(97)00147-2 9659995

[B36] Kwon S. Park H.-S. Stanley C. J. Kim J. Kim J. Damiano D. L. (2012). A practical strategy for semg-based knee joint moment estimation during gait and its validation in individuals with cerebral palsy. IEEE Trans. Biomed. Eng. 59 (5), 1480–1487. 10.1109/tbme.2012.2187651 22410952 PMC3594799

[B37] Laird P. Saul R. (1994). “Automated feature extraction for supervised learning,” in Proceedings of the First IEEE Conference on Evolutionary Computation. IEEE World Congress on Computational Intelligence (IEEE), 674–679.

[B38] Lim H. Kim B. Park S. (2020). Prediction of lower limb kinetics and kinematics during walking by a single imu on the lower back using machine learning. Sensors 20 (1), 130. 10.3390/s20010130 PMC6982819 31878224

[B39] Lu T.-W. O connor J. (1999). Bone position estimation from skin marker Co-ordinates using global optimisation with joint constraints. J. biomechanics 32 (2), 129–134. 10.1016/s0021-9290(98)00158-4 10052917

[B40] Luu T. P. Low K. Qu X. Lim H. Hoon K. (2014). An individual-specific gait pattern prediction model based on generalized regression neural networks. Gait posture 39 (1), 443–448. 10.1016/j.gaitpost.2013.08.028 24071020

[B41] Madgwick S. O. Harrison A. J. Vaidyanathan R. (2011). “Estimation of imu and marg orientation using a gradient descent algorithm,” in 2011 IEEE international conference on rehabilitation robotics (IEEE), 1–7. 10.1109/ICORR.2011.5975346 22275550

[B42] Mantoan A. Pizzolato C. Sartori M. Sawacha Z. Cobelli C. Reggiani M. (2015). Motonms: a Matlab toolbox to process motion data for neuromusculoskeletal modeling and simulation. Source code Biol. Med. 10 (1), 12–14. 10.1186/s13029-015-0044-4 26579208 PMC4647340

[B43] Moghadam S. M. Yeung T. Choisne J. (2023a). A comparison of machine learning models’ accuracy in predicting lower-limb joints’ kinematics, kinetics, and muscle forces from wearable sensors. Sci. Rep. 13 (1), 5046. 10.1038/s41598-023-31906-z 36977706 PMC10049990

[B44] Moghadam S. M. Yeung T. Choisne J. (2023b). The effect of imu sensor location, number of features, and window size on a random forest model’s accuracy in predicting joint kinematics and kinetics during gait. IEEE Sensors J. 23, 28328–28339. 10.1109/jsen.2023.3317366

[B45] Morbidoni C. Cucchiarelli A. Agostini V. Knaflitz M. Fioretti S. Di Nardo F. (2021). Machine-learning-based prediction of gait events from emg in cerebral palsy children. IEEE Trans. Neural Syst. Rehabilitation Eng. 29, 819–830. 10.1109/tnsre.2021.3076366 33909568

[B46] Mundt M. Johnson W. R. Potthast W. Markert B. Mian A. Alderson J. (2021). A comparison of three neural network approaches for estimating joint angles and moments from inertial measurement units. Sensors 21 (13), 4535. 10.3390/s21134535 34283080 PMC8271391

[B47] Mundt M. Thomsen W. Witter T. Koeppe A. David S. Bamer F. (2020). Prediction of lower limb joint angles and moments during gait using artificial neural networks. Med. Biol. Eng. Comput. 58 (1), 211–225. 10.1007/s11517-019-02061-3 31823114

[B48] Nolte D. Tsang C. K. Zhang K. Y. Ding Z. Kedgley A. E. Bull A. M. (2016). Non-linear scaling of a musculoskeletal model of the lower limb using statistical shape models. J. biomechanics 49 (14), 3576–3581. 10.1016/j.jbiomech.2016.09.005 PMC6399126 27653375

[B49] Onis M. d. Onyango A. W. Borghi E. Siyam A. Nishida C. Siekmann J. (2007). Development of a who growth reference for school-aged children and adolescents. Bull. World health Organ. 85 (9), 660–667. 10.2471/blt.07.043497 18026621 PMC2636412

[B50] Ren S. Wang W. Hou Z.-G. Chen B. Liang X. Wang J. (2019). Personalized gait trajectory generation based on anthropometric features using random forest. J. Ambient Intell. Humaniz. Comput. 14, 15597–15608. 10.1007/s12652-019-01390-3

[B51] Sabatini A. M. (2006). Quaternion-based extended kalman filter for determining orientation by inertial and magnetic sensing. IEEE Trans. Biomed. Eng. 53 (7), 1346–1356. 10.1109/tbme.2006.875664 16830938

[B52] Seifer A.-K. Küderle A. Dorschky E. Moradi H. Hannemann R. Eskofier B. (2023). Step length and gait speed estimation using a hearing aid integrated accelerometer: a comparison of different algorithms. 10.1109/JBHI.2024.3454824 39236137

[B53] Senden R. Marcellis R. Meijer K. Willems P. Lenssen T. Staal H. (2023). Dataset of 3d gait analysis in typically developing children walking at three different speeds on an instrumented treadmill in virtual reality. Data Brief 48, 109142. 10.1016/j.dib.2023.109142 37113500 PMC10126839

[B54] Sharifi Renani M. Eustace A. M. Myers C. A. Clary C. W. (2021). The use of synthetic imu signals in the training of deep learning models significantly improves the accuracy of joint kinematic predictions. Sensors 21 (17), 5876. 10.3390/s21175876 34502766 PMC8434290

[B55] Sharifi Renani M. Myers C. A. Zandie R. Mahoor M. H. Davidson B. S. Clary C. W. (2020). Deep learning in gait parameter prediction for oa and tka patients wearing imu sensors. Sensors 20 (19), 5553. 10.3390/s20195553 32998329 PMC7582246

[B56] Sivakumar S. Gopalai A. A. Lim K. H. Gouwanda D. (2019). Artificial neural network based ankle joint angle estimation using instrumented foot insoles. Biomed. Signal Process. Control 54, 101614. 10.1016/j.bspc.2019.101614

[B57] Slater A. A. Hullfish T. J. Baxter J. R. (2018). The impact of thigh and shank marker quantity on lower extremity kinematics using a constrained model. BMC Musculoskelet. Disord. 19 (1), 399–410. 10.1186/s12891-018-2329-7 30424811 PMC6234533

[B58] Slijepcevic D. Zeppelzauer M. Unglaube F. Kranzl A. Breiteneder C. Horsak B. (2023). Explainable machine learning in human gait analysis: a study on children with cerebral palsy. IEEE Access.

[B59] Stetter B. J. Krafft F. C. Ringhof S. Stein T. Sell S. (2020). A machine learning and wearable sensor based approach to estimate external knee flexion and adduction moments during various locomotion tasks. Front. Bioeng. Biotechnol. 8, 9. 10.3389/fbioe.2020.00009 32039192 PMC6993119

[B60] Stolze H. Kuhtz-Buschbeck J. Mondwurf C. Jöhnk K. Friege L. (1998). Retest reliability of spatiotemporal gait parameters in children and adults. Gait posture 7 (2), 125–130. 10.1016/s0966-6362(97)00043-x 10200382

[B61] Tan J.-S. Tippaya S. Binnie T. Davey P. Napier K. Caneiro J. (2022). Predicting knee joint kinematics from wearable sensor data in people with knee osteoarthritis and clinical considerations for future machine learning models. Sensors 22 (2), 446. 10.3390/s22020446 35062408 PMC8781640

[B62] Vigneron V. Dorizzi B. Khouri N. Desailly E. (2017). Predicting postoperative gait in cerebral palsy. Gait posture 52, 45–51. 10.1016/j.gaitpost.2016.11.012 27871017

[B63] Yeung T. Cantamessa A. Kempa-Liehr A. W. Besier T. Choisne J. (2023). Personalized machine learning approach to estimating knee kinematics using only shank-mounted IMU. IEEE Sensors J. 23, 12380–12387. 10.1109/jsen.2023.3267398

[B64] Zhang B. Zhang Y. Begg R. K. (2009). Gait classification in children with cerebral palsy by bayesian approach. Pattern Recognit. 42 (4), 581–586. 10.1016/j.patcog.2008.09.025

[B65] Zhang Y. Ma Y. (2019). Application of supervised machine learning algorithms in the classification of sagittal gait patterns of cerebral palsy children with spastic diplegia. Comput. Biol. Med. 106, 33–39. 10.1016/j.compbiomed.2019.01.009 30665140

