# Supplementary material for: 3D gait analysis in children using wearable sensors: feasibility of predicting joint kinematics and kinetics with personalized machine learning models and inertial measurement units
Source: Front Bioeng Biotechnol. 2024 Mar 20;12:1372669. doi: 10.3389/fbioe.2024.1372669 (PMC10987962; doi:10.3389/fbioe.2024.1372669)
Supplement: Supplementary file 1 [file DataSheet1.docx]

Supplementary Material

# Supplementary Figures and Tables

## Supplementary Tables

Supplementary Tabel A1: The mean Normalized Root Mean Square Error (NRMSE) between the outputs of OpenSim Inverse Kinematics (IK) and the Machine Learning (ML) model, accompanied by its corresponding standard deviation (SD), computed across all joints and motion planes.

|  | Average kinematics NRMSE (%) ± SD | |
| --- | --- | --- |
| Population | Intra-subject | Inter-subject |
| Younger TD children (≤ 10 years old) | 11.0 ± 2.1 | 20.1 ± 4.9 |
| Older TD children (> 10 years old) | 9.3 ± 1.6 | 19.8 ± 4.2 |

Supplementary Tabel A2: The mean NRMSE between the outputs of OpenSim Inverse Dynamics (ID) and the ML model, accompanied by its corresponding SD, computed across all joints and motion planes.

|  | Average kinetics NRMSE (%) ± SD | |
| --- | --- | --- |
| Population | Intra-subject | Inter-subject |
| Younger TD children (≤ 10 years old) | 11.5 ± 2.0 | 16.2 ± 4.8 |
| Older TD children (> 10 years old) | 10.5 ± 1.2 | 11.9 ± 1.1 |

## Supplementary Figures


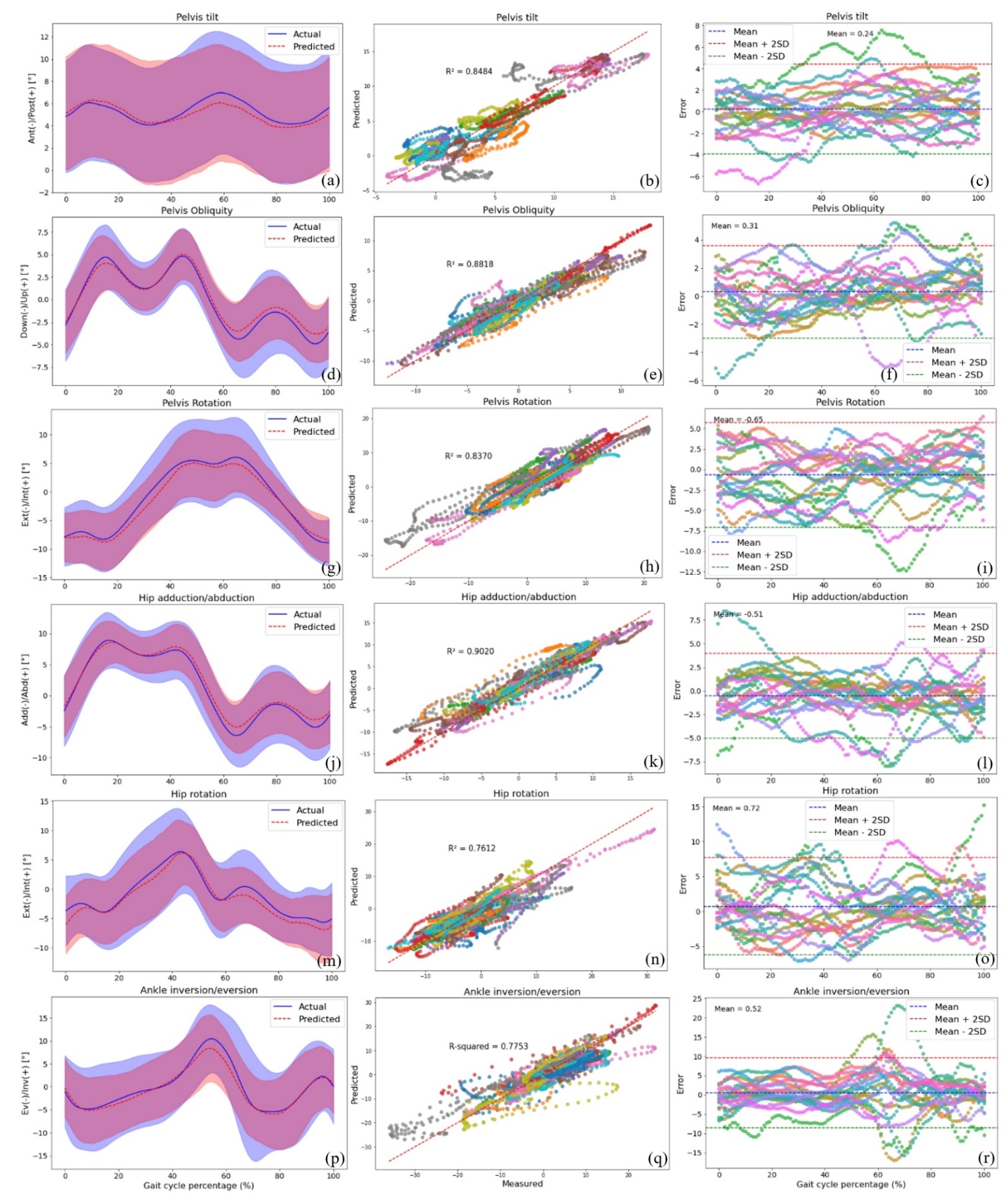


**Supplementary Figure A1:** The plots are made across all participants in the intra-subject examination, specifically for pelvis tilt (a, b, c), pelvis obliquity (d, e, f), pelvis rotation (g, h, i), hip adduction/abduction (j, k, l), hip rotation (m, n, o), and ankle inversion/eversion (p, q, r) joint angles. (a), (d), (g), (j), (m), and (p) present the RF model's average predictions (the dashed red line represents the average, and the red shaded area indicates the SD) for joint angles, utilizing data from IMUs placed on the feet. These predictions are compared to the joint angles derived from the OpenSim IK tool (the solid blue line represents the average, and the blue shaded area indicates the SD). Figures (b), (e), (h), (k), (n), and (q) illustrate the correlation and R-squared (R²) values for the mentioned joint angle targets. In (c), (f), (i), (l), (o), and (r), we utilized Bland-Altman plots to visually depict the errors throughout one gait cycle for all participants. In these plots, the dashed blue line represents the mean error, and the mean ± 2SD is depicted as dashed red and green lines. Each distinct color in these plots represents the results of one participant.


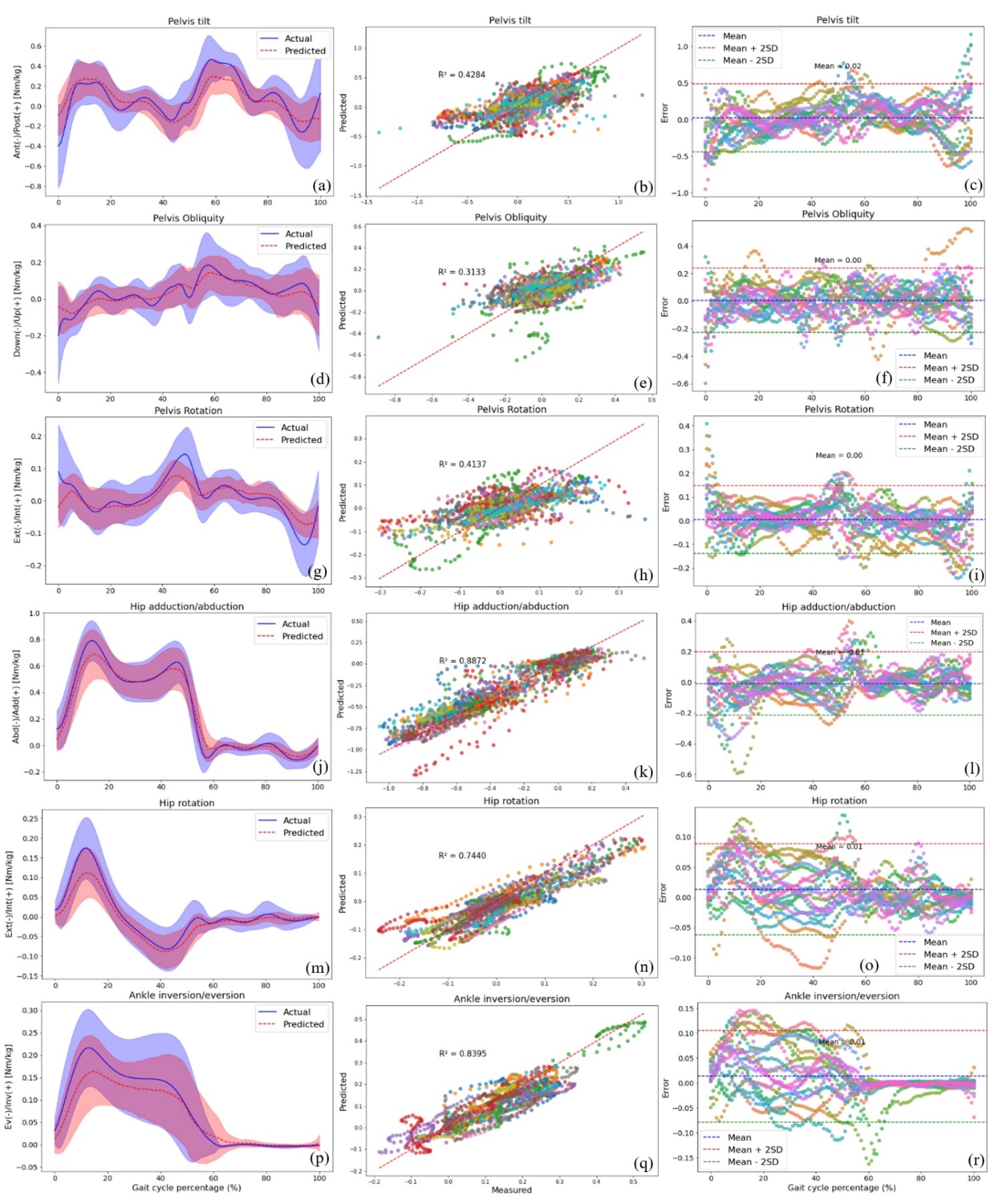


**Supplementary Figure A2:** The plots are made across all participants in the intra-subject examination, specifically for pelvis tilt (a, b, c), pelvis obliquity (d, e, f), pelvis rotation (g, h, i), hip adduction/abduction (j, k, l), hip rotation (m, n, o), and ankle inversion/eversion (p, q, r) joint moments. (a), (d), (g), (j), (m), and (p) present the RF model's average predictions (the dashed red line represents the average, and the red shaded area indicates the SD) for joint angles, utilizing data from IMUs placed on the feet. These predictions are compared to the joint angles derived from the OpenSim ID tool (the solid blue line represents the average, and the blue shaded area indicates the SD). Figures (b), (e), (h), (k), (n), and (q) illustrate the correlation and R-squared (R²) values for the mentioned joint angle targets. In (c), (f), (i), (l), (o), and (r), we utilized Bland-Altman plots to visually depict the errors throughout one gait cycle for all participants. In these plots, the dashed blue line represents the mean error, and the mean ± 2SD is depicted as dashed red and green lines. Each distinct color in these plots represents the results of one participant.
